# Sacubitril/valsartan improves all-cause mortality in heart failure patients with reduced ejection fraction and chronic kidney disease

**DOI:** 10.1007/s10557-022-07421-0

**Published:** 2023-01-07

**Authors:** Wei-Chieh Lee, Ting-Wei Liao, Tien-Yu Chen, Hsiu-Yu Fang, Yen-Nan Fang, Huang-Chung Chen, Yu-Sheng Lin, Shang-Hung Chang, Mien-Cheng Chen

**Affiliations:** 1 Institute of Clinical Medicine, College of Medicine, National Cheng Kung University, Tainan, Taiwan; 2 Division of Cardiology, Department of Internal Medicine, Chi Mei Medical Center, Tainan, Taiwan; 3 Division of Cardiology, Department of Internal Medicine, Kaohsiung Chang Gung Memorial Hospital, Chang Gung University College of Medicine, 123 Ta Pei Road, Niao Sung District Kaohsiung City, 83301 Taiwan; 4 School of Medicine, College of Medicine, National Sun Yat-sen University, Kaohsiung City, Taiwan; 5 Center for Big Data Analytics and Statistics, Chang-Gung University and Hospital, Taipei, Taiwan; 6 Division of Cardiology, Chang Gung Memorial Hospital, Chiayi, Taiwan

**Keywords:** Sacubitril/valsartan, Heart failure, Renal protection, Chronic kidney disease, All-cause mortality

## Abstract

**Background:**

Impaired renal function is frequently observed in patients with heart failure and reduced ejection fraction (HFrEF). The differential effect of sacubitril/valsartan and angiotensin-converting enzyme inhibitors/angiotensin-receptor blockers (ACEIs/ARBs) on the clinical and renal outcomes in patients with HFrEF and chronic kidney disease (CKD) remains unknown.

**Aims:**

This study aimed to explore the differential effect of sacubitril/valsartan and ACEI/ARB on the clinical and renal outcomes as well as renal function over a 12-month follow-up period in HFrEF patients with and without CKD.

**Methods:**

Patients with HfrEF (LVEF ≤35%) and NYHA class ≥II were enrolled from the Chang Gung Research Database between 2017 and 2020. Baseline characteristics were compared between patients prescribed sacubitril/valsartan and ACEI/ARB. After propensity score matching, the following clinical and renal outcomes were compared between the two groups in patients with and without CKD over a 12-month follow-up period: acute kidney injury (AKI), emergent dialysis/renal death, HF hospitalization, cardiovascular mortality, and all-cause mortality.

**Results:**

This study enrolled 3735 HFrEF patients with a mean left ventricular EF of 27.56 ± 5.86%, who had been prescribed sacubitril/valsartan (N = 1708) or ACEI/ARB (N = 2027). After propensity score matching, the clinical and renal outcomes did not differ between the sacubitril/valsartan and ACEI/ARB groups in patients without CKD. In patients with CKD, the ACEI/ARB group had a significantly higher incidence of all-cause mortality than the sacubitril/valsartan group (14.89% vs. 10.50%; hazard ratio 1.46; 95% confidence interval 1.06–2.00; *p* = 0.02), and the incidence of AKI, HF hospitalization, and CV mortality did not differ between the two groups.

**Conclusions:**

Sacubitril/valsartan had a lower all-cause mortality compared to ACEI/ARB in symptomatic HFrEF patients with CKD. Further prospective randomized studies are warranted to confirm our findings.

**Supplementary Information:**

The online version contains supplementary material available at 10.1007/s10557-022-07421-0.

## Background

The prevalence of heart failure (HF) in East Asian countries ranges from 1.3 to 6.7% and increases with age [1]. Several studies have shown that using angiotensin-converting enzyme inhibitors/angiotensin-receptor blockers (ACEIs/ARBs) for HF can reduce overall mortality by 16–40% [2–4]; therefore, ACEIs/ARBs are important guideline-directed medical therapy for symptomatic HF [5, 6]. In the PARADIGM-HF study, angiotensin receptor–neprilysin inhibitor, sacubitril/valsartan, reduced the risks of death and hospitalization for HF in patients with HF and reduced ejection fraction (HFrEF) when compared with enalapril [7]. Accordingly, the 2022 AHA/ACC/HFSA guideline recommends that if patients have chronic symptomatic HFrEF with NYHA class II or III symptoms and they tolerate an ACEI or ARB, they should be switched to sacubitril/valsartan because of improvement in echocardiographic parameters, morbidity, and mortality [8, 9].

Renal dysfunction is highly prevalent in patients with HF and is associated with poor outcomes [10]. The cardiorenal syndrome (CRS) encompasses a spectrum of disorders involving both the heart and kidneys in which acute or chronic dysfunction in one organ may induce acute or chronic dysfunction in the other organ, and the consensus conference of the Acute Dialysis Quality Initiative classifies CRS into five types: acute CRS, chronic CRS, acute renocardiac syndrome, chronic renocardiac syndrome, and secondary CRS [11, 12]. The proposed pathophysiological mechanisms of CRS are activation of the renin–angiotensin–aldosterone system (RAAS) axis, the sympathetic nervous system, and arginine vasopressin secretion, leading to fluid retention, increased preload, and worsening HF [12]. Although, the use of RAAS inhibition to slow down chronic kidney disease (CKD) progression is well established, there is a lack of data on long-term clinical and renal outcomes in patients with CKD from the trials of HF. Moreover, evidence for sacubitril/valsartan and ACEIs/ARBs in HFrEF patients with CKD or end stage renal disease remains lacking. Therefore, the differential effect of sacubitril/valsartan and ACEIs/ARBs on the clinical and renal outcomes in patients with HFrEF and CKD remains unknown.

Accordingly, we conducted a large cohort study to explore the differential effect of sacubitril/valsartan and ACEI/ARB on the clinical and renal outcomes as well as renal function over a 12-month follow-up period in HFrEF patients with and without CKD.

## Methods

### Patient population

We enrolled patients with HFrEF from January 2017 to December 2020, and their medical history, including detailed laboratory values, echocardiographic parameters, and drug use, were obtained from the Chang Gung Research Database (CGRD), the largest healthcare system in Taiwan.

The inclusion criteria were age ≥18 years, diagnosis of HF (International Classification of Diseases, Ninth Revision, Clinical Modification [ICD-9-CM] code 428.xx or Tenth Revision [ICD-10] code I50) with NYHA functional class ≥II, and left ventricular ejection fraction (LVEF) ≤35%, patients who had been prescribed sacubitril/valsartan or the same ACEIs/ARBs for at least half a year, and patients with baseline and follow-up renal function available. Patients who received sacubitril/valsartan or the same ACEIs/ARBs for less than half a year and those without baseline or following renal function were excluded. A flowchart of the study population is shown in Supplemental Fig. 1. Patients with LVEF ≤35% were enrolled in this study because the reimbursement criteria for sacubitril/valsartan in the Taiwan healthcare system during the study period were patients with LVEF ≤35% who remained symptomatic after optimally medical treatment with β-blocker and ACEIs or ARBs for at least 4 weeks.

Data on general demographics, comorbidities, baseline CrCl, LVEF, medication use, acute kidney injury (AKI), emergent dialysis or renal death, cardiovascular (CV) mortality, and all-cause mortality of patients were obtained. Echocardiographic examination was recommended to perform every 3–6 months for the patients with HFrEF. Baseline CrCl and body mass index were based on data within one month before using sacubitril/valsartan or ACEIs/ARBs.

### Ethical statement

This retrospective study conformed with the ethical guidelines of the 1975 Declaration of Helsinki and was approved for human research by the institutional review committee of Kaohsiung Chang Gung Memorial Hospital (number: 202100981B0C501).

### Definition

CKD was defined as a CrCl of <60 mL/min for >3 months, and the CrCl categories and CKD are as follows: stage 1 (normal or high, CrCl ≥90 mL/min), stage 2 (mildly decreased, CrCl of 60–89 mL/min), CKD 3a (mildly to moderately decreased, CrCl of 45–59 mL/min), CKD 3b (moderately to severely decreased, CrCl of 30–44 mL/min), CKD 4 (severely decreased, CrCl of 15–29 mL/min), and CKD 5 (CrCl of <15 mL/min, kidney failure with or without renal replacement therapy) [13]. Stage 1 and stage 2 were classified as non-CKD. End stage renal disease (ESRD) was defined as the need for renal replacement therapy including peritoneal dialysis, hemodialysis, or renal transplantation [13]. AKI was defined as an increase in serum Cr by ≥0.3 mg/dl within 48 h or increase in serum Cr to ≥1.5 times the baseline levels, which was known or presumed to have occurred within the prior seven days [14]. The need for hemodialysis was defined as a patient presenting with oliguria and anuria and undergoing hemodialysis. Renal death was defined as death where renal disease was the underlying cause of death. CV mortality was defined as death related to cardiovascular causes. All-cause mortality was defined as death due to any cause.

### Study endpoint

The study endpoints were AKI, emergent dialysis, renal death, HF hospitalization, CV mortality, and all-cause mortality.

### Statistical analyses

Data are presented as mean ± standard deviation or numbers (percentages). The clinical characteristics of the two groups were compared using the independent samples t-test and chi-square test for categorical variables. Propensity score matching was performed using a multivariate logistic regression model to adjust for differences in baseline characteristics (age, gender, mean LVEF, baseline CrCl, comorbidities, and medication) in the matched analysis. Using the estimated logits, the different groups had the closest estimated logit values for comparison between different groups. Matching quality was analyzed using the absolute value of the standardized mean difference between the groups after matching, where a value lower than 0.1 represented negligible difference between the groups. The incidences of AKI, HF hospitalization, CV mortality, and all-cause mortality during long-term follow-up were expressed with Kaplan–Meier survival curves and were compared by log-rank test. The risks of time to event outcomes between groups were compared using a Cox proportional hazards model. Statistical significance was set at *p* <0.05. All analyses were performed using SAS version 9.4 (SAS Institute. Inc., Cary, NC, USA).

## Results

### Baseline characteristics in the sacubitril/valsartan and ACEI/ARB groups before and after propensity score matching

This study enrolled 3735 participants, and their baseline characteristics and medication use are shown in Table 1. Before propensity score matching, older age, lower prevalence of male sex, and lower body mass index values were observed in the ACEI/ARB group (Table 1). Lower serum Cr and higher CrCl values were observed in the sacubitril/valsartan group. A higher prevalence of ivabradine, mineralocorticoid receptor antagonist, and diuretic use was observed in the sacubitril/valsartan group. Moreover, implantable cardioverter-defibrillator and cardiac resynchronization therapy (CRT) placement was more common in the sacubitril/valsartan group. The prevalence of ESRD with regular dialysis was 26.64% in the sacubitril/valsartan group and 29.16% in the ACEI/ARB group and did not differ between the two groups.

**Table 1:** Patients’ demographics before and after propensity score matching

	Before propensity score matching			After propensity score matching			
	Sacubitril/valsartan	ACEI/ARB	P value	Sacubitril/valsartan	ACEI/ARB	P value	SMD
Number	1708	2027		1251	1251		
General demographics							
Age (years)	61 (14.19)	65 (14.33)	<0.01	63 (13.87)	63 (14.52)	0.68	0.02
Male sex (%)	1313 (76.87)	1439 (70.99)	<0.01	942 (75.30)	943 (75.38)	0.96	<0.01
BMI (kg/m^2^)	26.02 (5.03)	25.24 (4.67)	<0.01	25.72 (4.81)	25.52 (4.69)	0.30	
Comorbidities							
Diabetes mellitus (%)	880 (51.52)	1074 (52.98)	0.37	689 (55.08)	646 (51.64)	0.08	
Hypertension (%)	1228 (71.9)	1493 (73.66)	0.23	929 (74.26)	902 (72.10)	0.22	
PAOD (%)	69 (4.04)	97 (4.79)	0.27	59 (4.72)	52 (4.16)	0.50	
COPD (%)	408 (23.89)	463 (22.84)	0.45	332 (26.54)	293 (23.42)	0.07	
CAD (%)	1156 (67.68)	1366 (67.39)	0.85	874 (69.86)	834 (66.67)	0.09	
ESRD with regular dialysis (%)	455 (26.64)	591 (29.16)	0.09	357 (28.54)	345 (27.58)	0.59	
Smoking (%)	511 (33.03)	560 (30.14)	0.07	360 (31.09)	380 (32.59)	0.43	
AF (%)	416 (24.36)	514 (25.36)	0.48	334 (26.70)	307 (24.54)	0.22	
Laboratory examination							
Creatinine (mg/dL)	1.69 (1.99)	1.94 (2.41)	<0.01	1.76 (2.06)	1.82 (2.21)	0.53	
Estimated CrCl (ml/min)	68.18 (43.77)	59.41 (39.00)	<0.01	64.3 (40.32)	63.6 (39.73)	0.66	0.02
Mean LVEF (%)	27.35 (5.67)	27.74 (6.02)	0.04	27.41 (5.62)	27.58 (5.90)	0.47	0.03
Medication							
β-blocker (%)	1468 (85.95)	1708 (84.26)	0.15	1071 (85.61)	1063 (84.97)	0.65	
Ivabradine (%)	383 (22.42)	247 (12.19)	<0.01	223 (17.83)	214 (17.11)	0.64	0.02
MRA (%)	920 (53.86)	719 (35.47)	<0.01	607 (48.52)	610 (48.76)	0.90	<0.01
Diuretic (%)	1011 (59.19)	988 (48.74)	<0.01	714 (57.07)	724 (57.87)	0.69	0.02
Device							
ICD+CRT (%)	94 (5.5)	58 (2.86)	<0.01	78 (6.24)	38 (3.04)	<0.01	

Data are expressed as mean (standard deviation) or as number (percentage).

Abbreviation: ACEI/ARB, angiotensin converting enzyme inhibitor/angiotensin receptor blocker; SMD, standardized mean difference; BMI, body mass index; PAOD, peripheral arterial occlusive disease; COPD, chronic obstructive pulmonary disease; CAD, coronary artery disease; ESRD, end stage renal disease; AF, atrial fibrillation; CrCl, creatinine clearance; LVEF, left ventricular ejection fraction; MRA, mineralocorticoid receptor antagonist; ICD, implantable cardioverter-defibrillator; CRT, cardiac resynchronization therapy

After propensity score matching, the prevalence of *implantable cardioverter-defibrillator*/CRT implantation remained higher in the sacubitril/valsartan group (6.24 % vs. 3.04 %; *p* < 0.01) (Table 1). The mean LVEF and renal function were similar between the two groups. The prevalence of ESRD with regular dialysis was 28.54% in the sacubitril/valsartan group and 27.58% in the ACEI/ARB group and did not differ between the two groups.

### Demographics of patients with and without CKD after propensity score matching

The baseline characteristics of patients without CKD after propensity score matching are listed in Table 2 and did not differ between the sacubitril/valsartan and ACEI/ARB groups. The baseline characteristics of patients with CKD after propensity score matching are listed in Table 3. The prevalence of implantable cardioverter-defibrillator/CRT device implantation remained higher in the sacubitril/valsartan group. In patients with CKD after propensity score matching, the prevalence of ESRD with regular dialysis was 58.62% in the sacubitril/valsartan group and 59.40% in the ACEI/ARB group and did not differ between the two groups.

**Table 2:** The demographics of patients without CKD after propensity score matching

	Sacubitril/valsartan	ACEI/ARB	P value	SMD
Number	736	736		
General demographics				
Age (years)	59.57 (13.4)	59.29 (14.04)	0.69	0.02
Male sex (%)	568 (77.17)	577 (78.40)	0.57	0.03
BMI (kg/m^2^)	26.26 (5.01)	25.83 (4.71)	0.11	0.09
Comorbidities				
Diabetes mellitus (%)	320 (43.48)	314 (42.66)	0.75	0.02
Hypertension (%)	471 (63.99)	461 (62.64)	0.59	0.03
PAOD (%)	14 (1.90)	19 (2.58)	0.38	0.05
COPD (%)	156 (21.20)	146 (19.84)	0.52	0.03
CAD (%)	464 (63.04)	461 (62.64)	0.87	0.01
Smoking (%)	234 (36.34)	237 (36.24)	0.97	<0.01
AF (%)	171 (23.23)	166 (22.55)	0.76	0.02
Laboratory examination				
Creatinine (mg/dL)	0.92 (0.18)	0.92 (0.20)	0.69	0.02
Estimated CrCl (ml/min)	88.66 (38.43)	87.54 (36.93)	0.59	0.03
Mean LVEF (%)	27.62 (5.54)	27.75 (5.91)	0.67	0.02
Medication				
β-blocker (%)	642 (87.23)	624 (84.78)	0.18	0.07
Ivabradine (%)	120 (16.3)	118 (16.03)	0.89	0.01
MRA (%)	375 (50.95)	372 (50.54)	0.88	0.01
Diuretic (%)	365 (49.59)	360 (48.91)	0.79	0.01
Device				
ICD+CRT (%)	29 (3.94)	20 (2.72)	0.19	0.07

Data are expressed as mean (standard deviation) or as number (percentage).

Abbreviation: CKD, chronic kidney disease; CrCl, creatinine clearance; ACEI/ARB, angiotensin converting enzyme inhibitor/angiotensin receptor blocker; SMD, standardized mean difference; BMI, body mass index; PAOD, peripheral arterial occlusive disease; COPD, chronic obstructive pulmonary disease; CAD, coronary artery disease; ESRD, end stage renal disease; AF, atrial fibrillation; LVEF, left ventricular ejection fraction; MRA, mineralocorticoid receptor antagonist; ICD, implantable cardioverter–defibrillator; CRT, cardiac resynchronization therapy

**Table 3:** The demographics of patients with CKD after propensity score matching

	Sacubitril/valsartan	ACEI/ARB	P value	SMD
Number	638	638		
General demographics				
Age (years)	67.32 (12.94)	67.23 (13.71)	0.91	0.01
Male sex (%)	462 (72.41)	463 (72.57)	0.95	<0.01
BMI (kg/m^2^)	25.19 (4.44)	25.24 (4.75)	0.85	0.01
Comorbidities				
Diabetes mellitus (%)	412 (64.58)	399 (62.54)	0.45	0.04
Hypertension (%)	537 (84.17)	531 (83.23)	0.65	0.03
PAOD (%)	45 (7.05)	48 (7.52)	0.75	0.02
COPD (%)	186 (29.15)	167 (26.18)	0.23	0.07
CAD (%)	481 (75.39)	447 (70.06)	0.03	0.12
ESRD with regular dialysis (%)	374 (58.62)	379 (59.40)	0.78	0.02
Smoking (%)	161 (26.88)	171 (27.85)	0.70	0.02
AF (%)	188 (29.47)	173 (27.12)	0.35	0.05
Laboratory examination				
Creatinine (mg/dL)	2.62 (2.65)	2.90 (3.09)	0.08	0.09
Estimated CrCl (ml/min)	38.83 (25.04)	37.60 (25.25)	0.40	0.05
Mean LVEF (%)	27.74 (5.31)	27.56 (5.77)	0.57	0.03
Medication				
β-blocker (%)	536 (84.01)	541 (84.80)	0.70	0.02
Ivabradine (%)	112 (17.55)	104 (16.30)	0.55	0.03
MRA (%)	290 (45.45)	287 (44.98)	0.87	0.01
Diuretic (%)	409 (64.11)	413 (64.73)	0.82	0.01
Device				
ICD+CRT (%)	44 (6.90)	22 (3.45)	0.01	0.16

Data are expressed as mean (standard deviation) or as number (percentage).

Abbreviation: CKD, chronic kidney disease; CrCl, creatinine clearance; ACEI/ARB, angiotensin converting enzyme inhibitor/angiotensin receptor blocker; SMD, standardized mean difference; BMI, body mass index; PAOD, peripheral arterial occlusive disease; COPD, chronic obstructive pulmonary disease; CAD, coronary artery disease; ESRD, end stage renal disease; AF, atrial fibrillation; LVEF, left ventricular ejection fraction; MRA, mineralocorticoid receptor antagonist; ICD, implantable cardioverter-defibrillator; CRT, cardiac resynchronization therapy

### Change in CrCl during one-year follow-up period in patients with and without CKD after propensity score matching

**Fig. 1:**
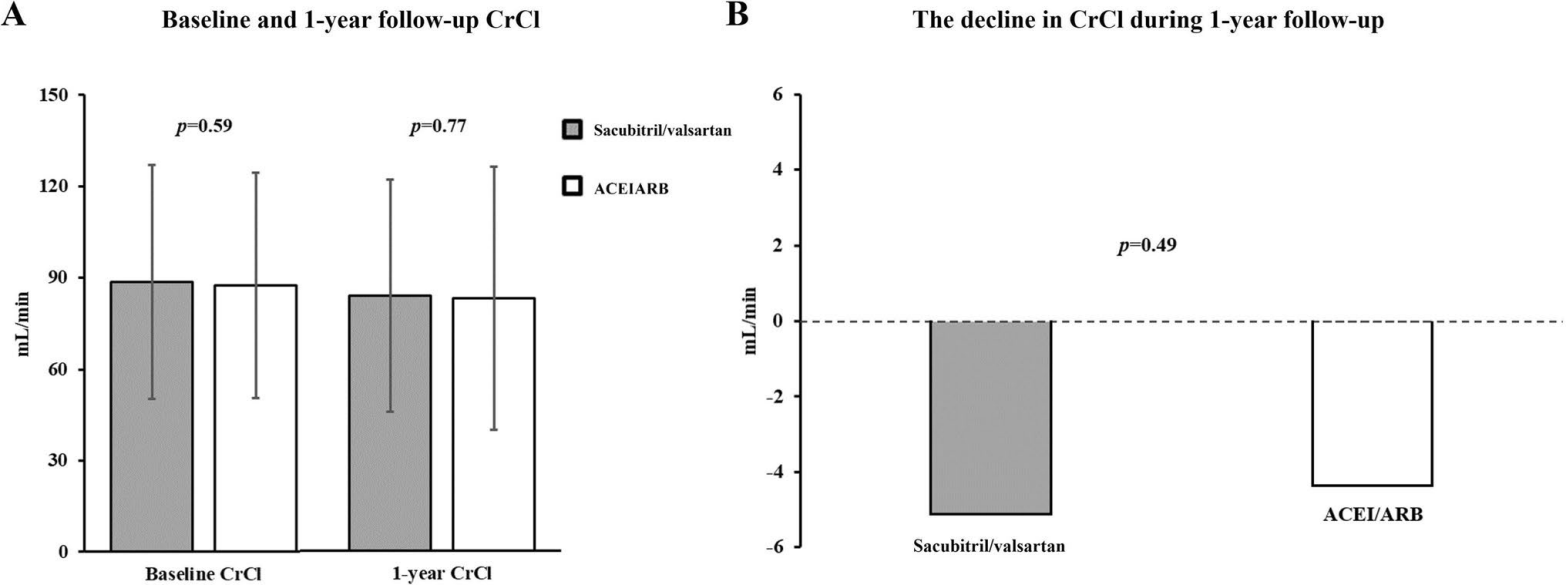
The difference in the baseline and 1-year follow-up CrCl values (**A**) and the decline in CrCl level (**B**) between the sacubitril/valsartan and ACEI/ARB groups in patients without chronic kidney disease after propensity score matching. CrCl, creatinine clearance; ACEI, angiotensin-converting enzyme inhibitor; ARB, angiotensin-receptor blocker

**Fig. 2:**
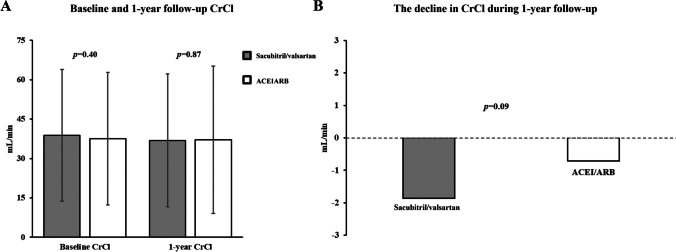
The difference in the baseline and 1-year follow-up CrCl values (**A**) and the decline in CrCl level (**B**) between the sacubitril/valsartan and ACEI/ARB groups in patients with chronic kidney disease after propensity score matching. CrCl, creatinine clearance; ACEI, angiotensin-converting enzyme inhibitor; ARB, angiotensin-receptor blocker

After propensity score matching, the decline in CrCl did not differ between the sacubitril/valsartan and ACEI/ARB groups in patients without CKD (Fig. 1) or with CKD (Fig. 2).

### One-year clinical and renal outcomes between sacubitril/valsartan and ACEI/ARB groups in the total study population and in patients with and without CKD after propensity score matching

In the total study population, the ACEI/ARB group had a significantly higher incidence of all-cause mortality than the sacubitril/valsartan group (sacubitril/valsartan vs. ACEI/ARB; 10.15% vs. 7.83%; hazard ration (HR) 1.31, 95% confidence interval (CI) 1.01–1.71; p = 0.04) (Table 4).

**Table 4:** Clinical outcomes and hazard ratio between ACEI/ARB and sacubitril/valsartan groups after propensity score matching

	Sacubitril/valsartan	ACEI/ARB	P value	HR	95% CI	P value
Total population						
AKI (%)	80 (6.39)	92 (7.35)	0.34	1.16	0.87–1.56	0.32
Emergent dialysis/renal death (%)	4 (0.32)	5 (0.40)	0.74	1.27	0.34–4.73	0.72
HF hospitalization (%)	210 (16.79)	194 (15.51)	0.38	0.94	0.77–1.14	0.50
CV mortality (%)	36 (2.88)	44 (3.52)	0.36	1.24	0.80–1.93	0.34
All-cause mortality (%)	98 (7.83)	127 (10.15)	0.04	1.31	1.01–1.71	0.04
Patients without CKD						
AKI (%)	23 (3.13)	26 (3.53)	0.66	1.13	0.64–2.00	0.67
Emergent dialysis/renal death (%)	0 (0.00)	0 (0.00)	1.00	–	–	–
HF hospitalization (%)	81 (11.01)	77 (10.46)	0.74	0.95	0.69–1.31	0.77
CV mortality (%)	15 (2.04)	13 (1.77)	0.70	0.87	0.41–1.83	0.71
All-cause mortality (%)	38 (5.16)	38 (5.16)	1.00	1.00	0.63–1.58	1.00
Patients with CKD						
Emergent dialysis/renal death (%)	4 (0.63)	5 (0.78)	1.00	1.30	0.35–4.83	0.70
HF hospitalization (%)	133 (20.85)	112 (17.55)	0.14	0.86	0.68–1.10	0.24
CV mortality (%)	24 (3.76)	33 (5.17)	0.22	1.42	0.83–2.43	0.20
All-cause mortality (%)	67 (10.50)	95 (14.89)	0.02	1.46	1.06–2.00	0.02

Data are expressed as mean (standard deviation) or as number (percentage).

Abbreviation: HR, hazard ratio; ACEI/ARB, angiotensin converting enzyme inhibitor/angiotensin receptor blocker; CI, confidence interval; AKI, acute kidney injury; HF, heart failure; CV, cardiovascular; CKD, chronic kidney disease

**Fig. 3:**
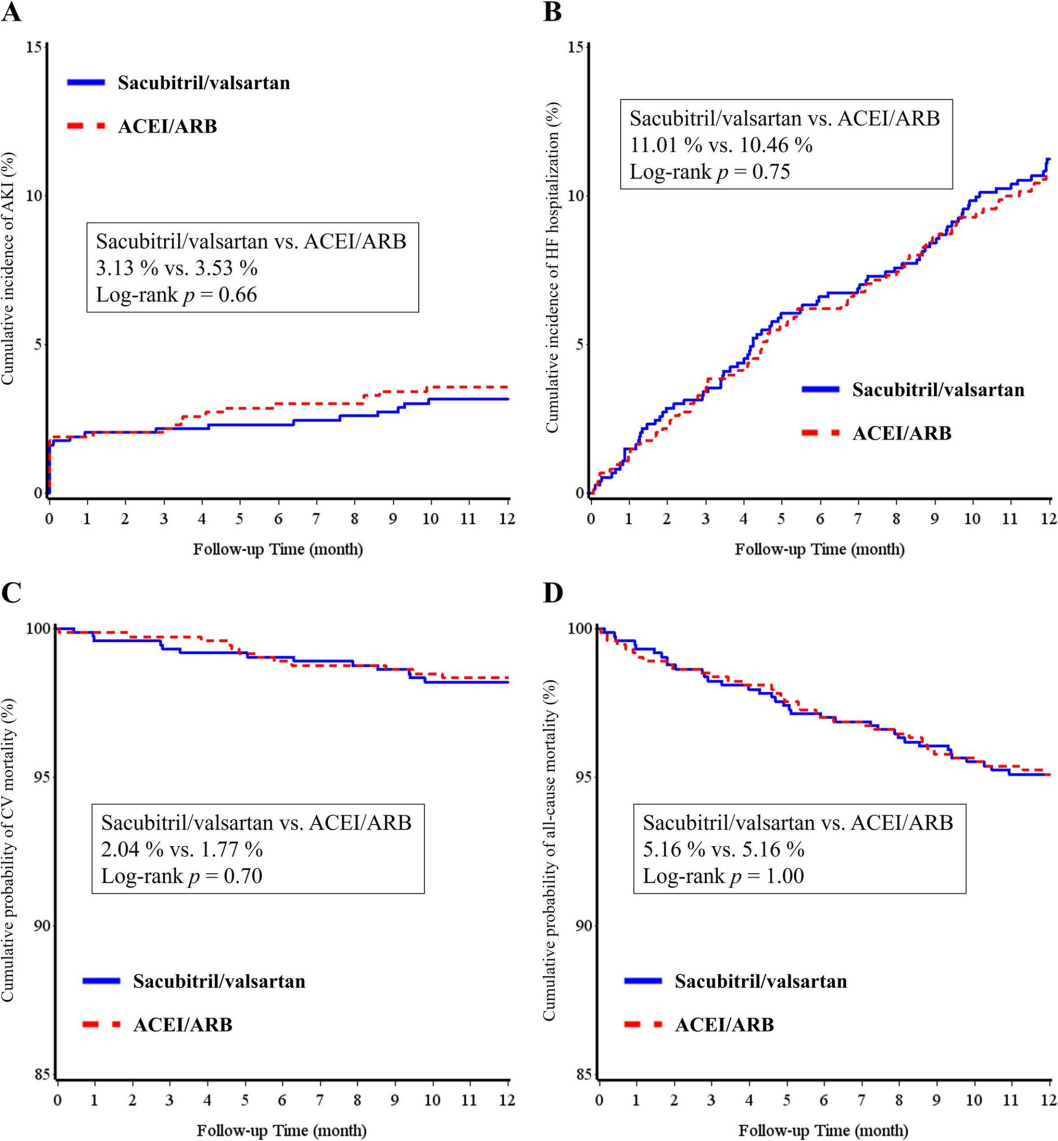
The Kaplan–Meier survival curves of AKI (**A**), HF hospitalization (**B**), CV mortality (**C**) and all-cause mortality (**D**) between the sacubitril/valsartan and ACEI/ARB groups in patients without chronic kidney disease after propensity score matching. ACEI, angiotensin-converting enzyme inhibitor; ARB, angiotensin-receptor blocker

In patients without CKD, the clinical and renal outcomes did not differ between the sacubitril/valsartan and ACEI/ARB groups (Table 4). The Kaplan–Meier survival curves of AKI, HF hospitalization, CV mortality, and all-cause mortality are shown in Fig. 3.

**Fig. 4:**
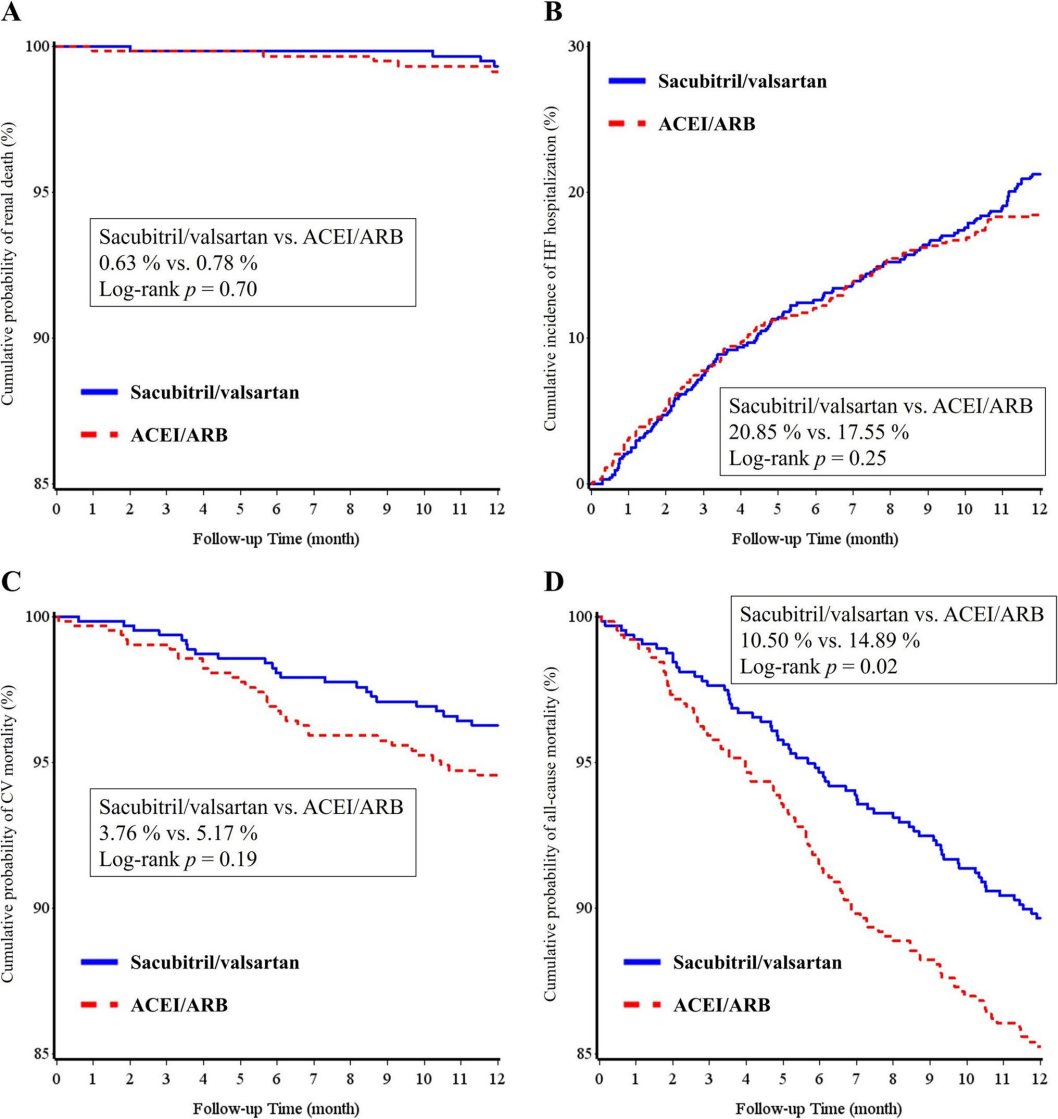
The Kaplan–Meier survival curves of renal death (**A**), HF hospitalization (**B**), CV mortality (**C**), and all-cause mortality (**D**) between the sacubitril/valsartan and ACEI/ARB groups in patients with chronic kidney disease after propensity score matching. ACEI, angiotensin-converting enzyme inhibitor; ARB, angiotensin-receptor blocker

In patients with CKD, the ACEI/ARB group had a significantly higher incidence of all-cause mortality than the sacubitril/valsartan group (14.89% vs. 10.50%; HR 1.46; 95% CI 1.06–2.00; *p* = 0.02). The incidence of emergent dialysis/renal death, HF hospitalization, and CV mortality did not differ between the two groups (Table 4). The Kaplan–Meier survival curves of renal death, HF hospitalization, CV mortality, and all-cause mortality are shown in Fig. 4. Significantly lower incidence of all-cause mortality was noted in the sacubitril/valsartan group when compared with the ACEI/ARB group (log-rank *p* value = 0.02).

## Discussion

In this large cohort study, the sacubitril/valsartan group had lower all-cause mortality compared to the ACEI/ARB group in HFrEF patients with CKD, but not in patients without CKD during the one-year follow-up period. The renal outcomes, AKI, and emergent dialysis/renal death did not differ between the sacubitril/valsartan and ACEI/ARB groups after propensity score matching. Moreover, there was no significant difference in the decline in CrCl between the two groups during the one-year follow-up period.

Sacubitril enhances the activity of natriuretic peptides via preventing the breakdown of natriuretic peptides, producing natriuresis, diuresis, and vasodilation, and ARBs inhibit the activated RAAS, and therefore, sacubitril/valsartan plays an important role in RAAS activated conditions, such as HF and CKD [14]. In an animal model of diabetes, sacubitril/valsartan elicited a renoprotective effect against overt proteinuria and reduced tubulointerstitial fibrosis [15]. Another animal study demonstrated that the renoprotective effect of sacubitril/valsartan was independent of antihypertensive efficacy, renal hemodynamics, or inflammation but might be related to the protective effect of natriuretic peptides on podocyte, which reduces associated glomerulosclerosis [16, 17]. Taken together, these animal study results suggest that sacubitril/valsartan could be an effective drug for renal protection.

One observational study showed that sacubitril/valsartan improved renal function after 12 months in patients with HFrEF, especially in subjects aged < 65 years and patients with CKD [18]. In the post-hoc analysis of PARADIGM-HF study, sacubitril/valsartan led to a slower rate of decrease in the estimated glomerular filtration rate, even in patients with CKD, despite causing a modest increase in urinary albumin/creatinine ratio [17]. Moreover, the benefits of sacubitril/valsartan therapy over enalapril on the primary outcome of a composite of death from cardiovascular causes or a first hospitalization for HF were maintained independently from urinary albumin/creatinine ratio increase or decrease [19]. Furthermore, a 25% increase in urinary albumin/creatinine ratio was associated with a higher risk of the renal composite endpoint in the enalapril arm, but not in the sacubitril/valsartan arm [19]. However, in the UK HARP-III trial, sacubitril/valsartan was found to have similar results on kidney function and albuminuria to irbesartan over a 12-month follow-up period in people with CKD [20]. Of note, no large randomized controlled study or large cohort study has been specifically conducted to explore the differential effect of sacubitril/valsartan and ACEI/ARB on the clinical and renal outcomes in patients with HFrEF and CKD.

In our study, sacubitril/valsartan therapy led to a lower all-cause mortality than ACEI/ARB therapy in symptomatic HFrEF patients with CKD, but not in patients without CKD, nearly 30% of the study patients having ESRD. Moreover, sacubitril/valsartan therapy led to a similar decline in CrCl compared to ACEI/ARB therapy during the one-year follow-up period. This study provided important data to fill the gap, in terms of all-cause mortality, in symptomatic patients with HFrEF and CKD.

### Study limitations

This study has several limitations. First, it is a retrospective cohort and nonrandomized study and we could not rule out bias in our study. Second, some unmeasured confounding factors and nephrotoxic agents might exist despite propensity score matching. Third, the severities of HF, including the value of N-terminal pro B-type natriuretic peptide and New York Heart Association functional classification during follow-up, were not available in this cohort study. Fourth, most patients received ACEI/ARB for at least one month in the sacubitril/valsartan group due to the reimbursement requirement of our healthcare system. Fifth, we enrolled symptomatic HF patients with LVEF ≤35% according to the reimbursement criteria for sacubitril/valsartan in the Taiwan healthcare system. Sixth, diagnosis of HF by the ICD-9M and ICD-10M codes relied on the physician’s decision, we also analyzed the medications consistent with the diagnosis of HF in the enrolled patients.

## Conclusions

Sacubitril/valsartan therapy had a lower all-cause mortality compared to ACEI/ARB therapy in symptomatic HFrEF patients with CKD over a 12-month follow-up period. Sacubitril/valsartan therapy had a similar decline in creatinine clearance compared to ACEI/ARB therapy in patients with and without CKD. Further large randomized studies are warranted to validate our findings.

### Supplementary information

ESM 1 (JPG 708 kb)

## Data Availability

The study data are available from the corresponding author upon reasonable request. This study was based, in part, on data from the CGRD provided by the Chang Gung Memorial Hospital. The interpretation and conclusions contained herein do not represent the positions of the Chang Gung Memorial Hospital.
