# Supplementary figures and images for: Sacubitril/valsartan improves all-cause mortality in heart failure patients with reduced ejection fraction and chronic kidney disease
Source: Cardiovasc Drugs Ther. 2023 Jan 7;38(3):505–15. doi: 10.1007/s10557-022-07421-0 (PMC11101538; doi:10.1007/s10557-022-07421-0)

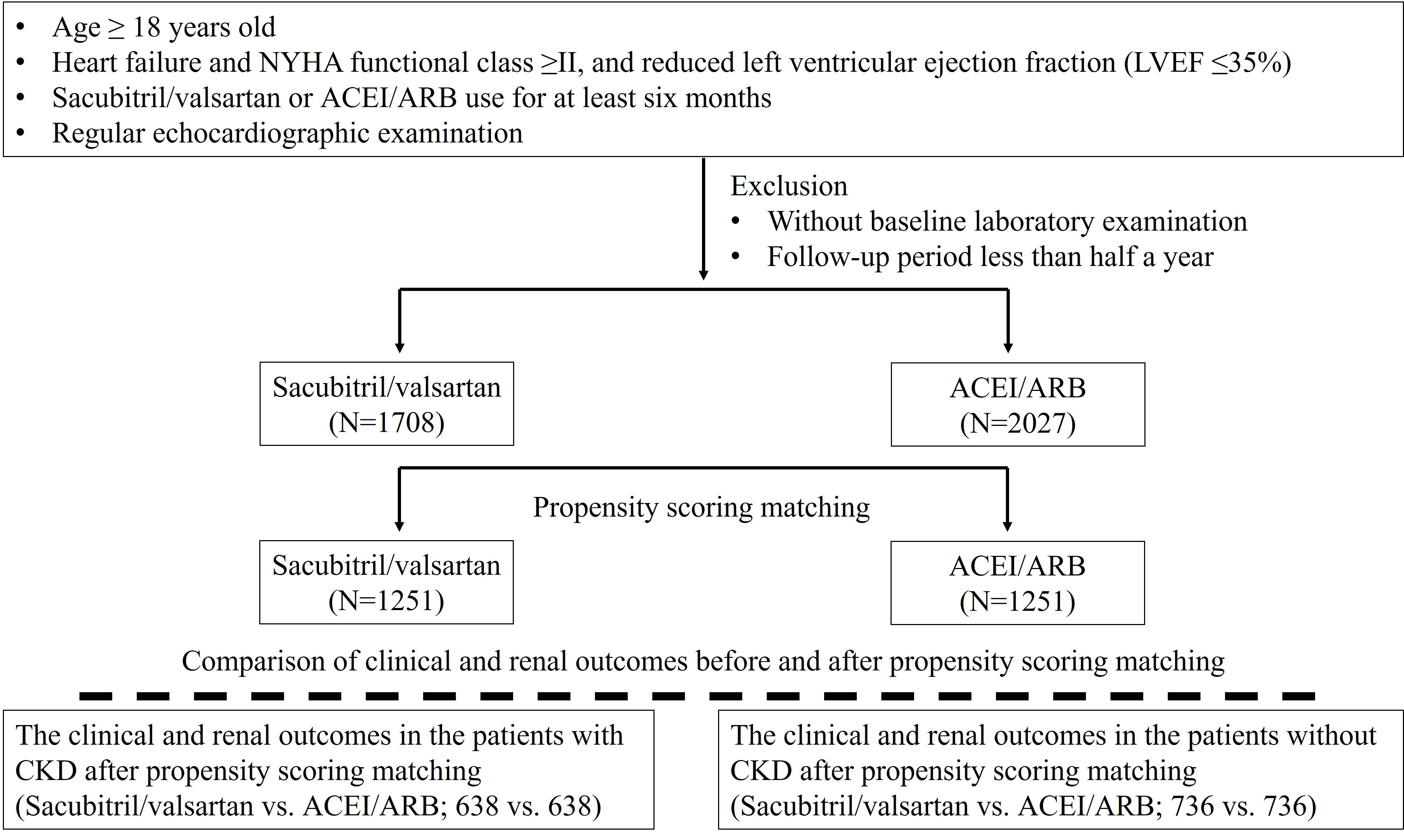

Supplement: Supplementary file 1 — (JPG 708 kb) [file 10557_2022_7421_MOESM1_ESM.jpg]
